# SRPassing Co-translational Targeting: The Role of the Signal Recognition Particle in Protein Targeting and mRNA Protection

**DOI:** 10.3390/ijms22126284

**Published:** 2021-06-11

**Authors:** Morgana K. Kellogg, Sarah C. Miller, Elena B. Tikhonova, Andrey L. Karamyshev

**Affiliations:** Department of Cell Biology and Biochemistry, Texas Tech University Health Sciences Center, Lubbock, TX 79430, USA; Morgana.Kellogg@ttuhsc.edu (M.K.K.); Sarah.C.Miller@ttuhsc.edu (S.C.M.); Elena.Tikhonova@ttuhsc.edu (E.B.T.)

**Keywords:** ribosome, mRNA translation, signal recognition particle (SRP), protein targeting and transport, protein sorting, signal sequence, protein quality control, translational control

## Abstract

Signal recognition particle (SRP) is an RNA and protein complex that exists in all domains of life. It consists of one protein and one noncoding RNA in some bacteria. It is more complex in eukaryotes and consists of six proteins and one noncoding RNA in mammals. In the eukaryotic cytoplasm, SRP co-translationally targets proteins to the endoplasmic reticulum and prevents misfolding and aggregation of the secretory proteins in the cytoplasm. It was demonstrated recently that SRP also possesses an earlier unknown function, the protection of mRNAs of secretory proteins from degradation. In this review, we analyze the progress in studies of SRPs from different organisms, SRP biogenesis, its structure, and function in protein targeting and mRNA protection.

## 1. Introduction

Cells have a very complex network of a number of cellular organelles surrounded by different membranes. To maintain their viability, cells constantly transport proteins from the place of their synthesis to these organelles or outside of the cells. Cell proteomics predicts that there are more than 10^9^ proteins in a single human cell [1]. Therefore, directing proteins to their correct destination is an important process for all cells. The correct identification and targeting of proteins are vital to ensure that the proteins are accurately folded, active, and delivered to the right place at the right time with the precise amount of protein needed. The cost of mistargeting, however, is large; many diseases are attributed to the aberrant localization of proteins, which results in cytotoxic aggregation, degradation, loss of expression, and misfolding of proteins. The cells have evolved several mechanisms and many specific protein targeting signals to ensure fast, efficient, and accurate targeting of proteins to prevent these problems. In this review, we briefly discuss different protein targeting signals and then focus on one of the major secretory pathways, the signal recognition particle (SRP) pathway, SRP biogenesis, SRP structure, and SRP cellular function.

## 2. Protein Targeting Signals

**Figure 1:**
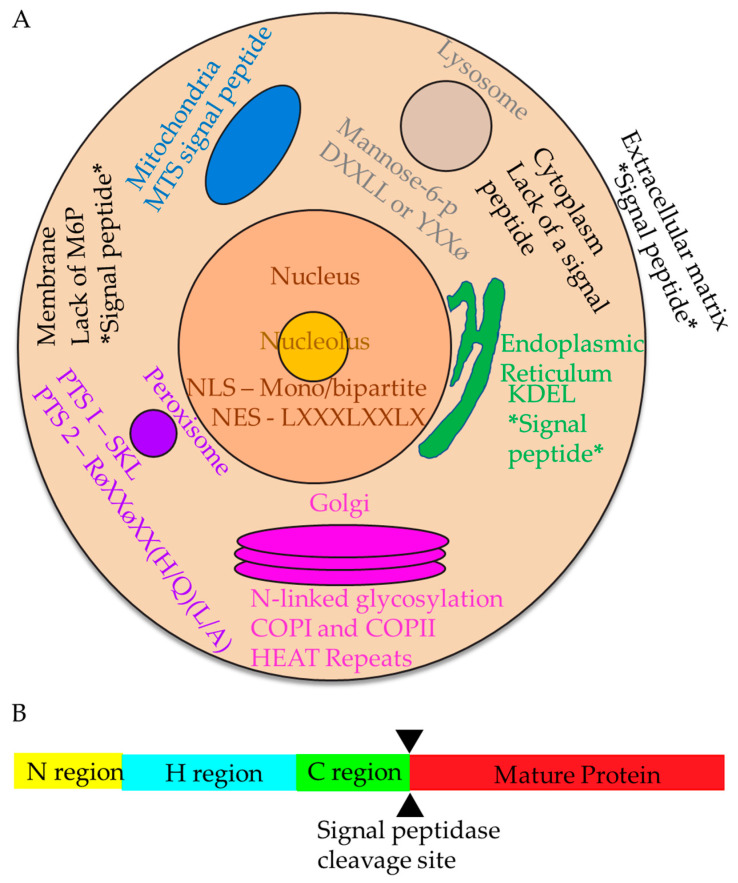
Cellular organelles and protein targeting signals in the mammalian cell. (**A**) Protein targeting signals. Within the cell, there are many different signals that direct proteins for proper localization. Nuclear (salmon circle) and nucleolar (yellow circle) signals include the NLS and NES. Signals to the endoplasmic reticulum include the signal peptide and KDEL, which prevents secretion from the ER. The cytoplasmic signals (light tan) are generally not well-defined and include everything that does not contain a signal peptide. Peroxisome (purple circle) signals are PTS1 and PTS2. The Golgi (magenta stacked ovals) signals include N-linked glycosylations, resident proteins are retained via HEAT repeats, and errant proteins are recaptured with COPI and COPII vesicles. Targeting signals to the lysosome (gray circle) include phosphorylated mannose, or DXXLL or YXXφ. Post-translational targeting to the plasma membrane (black outline) uses the absence of mannose-6-p, while co-translational signals include the signal peptide. Mitochondria (blue) have their own mitochondrial targeting signal peptide. Extracellular matrix-bound proteins (white background) are targeted using a signal peptide. All co-translational mechanisms of targeting are designated by two stars flanking the signal (e.g., *signal peptide*). (**B**) Diagram of a protein with a signal sequence. A signal sequence features a positive N-region (yellow), a hydrophobic (H) middle region (blue), a C-region (green), and a region that will become the mature protein once signal peptidase (cleavage site demarcated by black triangles) cleaves off the signal sequence (red).

Proteins contain information about subcellular localization in the form of special sequences for their transport. Cells rely on the recognition of these specific signals (localization sequences) embedded in the structure of polypeptide chains to sort proteins to their appropriate cellular compartments. This recognition, targeting, and transport can occur during or after protein synthesis at the ribosome, and these pathways are referred to as co-translational and post-translational processes, respectively, shown in Figure 1A. Targeting to mitochondria, the nucleus, and peroxisomes occurs mostly post-translationally, while protein targeting and importing into the endoplasmic reticulum (ER) occurs co-translationally, directed by its cytosolic targeting factor, SRP, the signal recognition particle [2,3,4,5,6,7].

Post-translational targeting includes signals to the peroxisome, nuclear entrance and exit signals, Golgi membrane localization, signals to the lysosome, and mitochondrial signal peptides. Many post-translational targeting features have been characterized. Peroxisomes have two signals: peroxisome targeting signal 1 (PTS1) and 2 (PTS2). The most common PTS1 signal is a serine-lysine-leucine at the carboxy-end [8] and is recognized by the receptor encoded by PEX5 on the peroxisomal membrane [9]. PTS2 uses the PEX5L and PEX7 receptors in mammals for transport into the peroxisome [10]. Nuclear targeting signals have both entry and exit codes: a nuclear localization signal (NLS) to target the nucleus and a nuclear exit signal (NES) to leave. Nuclear localization signals have a general consensus sequence featuring positively charged amino acids with linker regions in between, called a bipartite signal [11]. Importins, either by themselves or with cofactors, recognize NLSs to permit proteins to enter the nucleus. Nuclear exit signals consist of four hydrophobic residues with spacers between; the most common exit signal is LxxxLxxLxL, where x is any other amino acid, and L is leucine [12]. Exportins interact with the NES to permit proteins to leave the nucleus. The Golgi localization signal (GLS) consists of an N-terminal signal with HEAT motif (which stands for Huntingtin, elongation factor 3, protein phosphatase 2A, and yeast kinase TOR1) repeats, a motif consisting of two α helices with a short loop spacer region [13], and is at least 42 amino acid residues long [14]. The Trans-Golgi network, a key sorting step for secreted proteins, uses COPI- and COPII-lined vesicles to correctly target ER-retained and Golgi-retained proteins [15]. Lysosomal proteins are targeted by either a tyrosine-based motif, Yxxø, where Y is tyrosine and ø is a bulky hydrophobic residue, or a dual leucine motif, DxxLL, where D is aspartate (reviewed in [16]). Lysosomal proteins are modified with mannose-6-phosphate (M6P), which is recognized by M6P receptors on the Golgi membrane to be targeted to the endo-lysosomal pathway. Mitochondrial targeting signals (MTS) have no sequence homology, vary in length, are rich in basic residues at the amino terminus, and form amphipathic helices [17,18,19]. MTSs are recognized by translocase of outer membrane (TOM) and translocase of inner membrane (TIM) proteins to translocate mitochondrial-specific proteins correctly.

Targeting signals to the endoplasmic reticulum consist of two categories: signal recognition particle (SRP)-dependent, co-translational targeting (reviewed in [2]) and SRP-independent, post-translational targeting. SRP-independent targeting in mammals includes small secretory proteins [20,21,22], tail-anchored proteins [23,24], and other proteins not efficiently recognized by SRP [25,26]. Proteins retained in the ER have a lysine, aspartate, glutamate, leucine (KDEL) motif at the carboxy-end, which prevents further transport from the ER [27].

In contrast to post-translational pathways, co-translational targeting to the endoplasmic reticulum requires the signal recognition particle (SRP). The signal recognition particle recognizes N-terminal signal sequences of precursors of the secretory proteins. However, signal sequences do not have strong sequence homology at the amino acid residue level, and SRP does not recognize any specific sequence of amino acid residues. Instead, signal sequences are distinguished by three physicochemical characteristics: a positively charged N-terminus or N domain, H domain or hydrophobic region, and C-terminus with cleavage site for signal peptidase (Figure 1B) [28,29]. Additionally, signal peptidase cleavage sites are characterized by the “−3, −1 rule” [29,30]. Yet these same physicochemical characteristics are present in mitochondrial targeting sequences and can be localized to either mitochondria or ER; how does the cell distinguish between the two compartments? SRP has been shown to mistarget cytochrome oxidase subunit 1 to the ER when its nascent chain is exposed to the cytoplasm due to its ambiguous signal sequence [31]. In order to increase mitochondrial specificity, these ambiguous signal sequences are often modified with myristoyl or have a second start codon to produce a protein that decreases the affinity of the nascent chain for SRP [32,33]. These signal sequences then correctly target the mitochondria instead of the ER by SRP.

Around 30% of all proteins translocate to the ER using a co-translational pathway [34,35,36,37]. SRP-dependent translocation is the major route for co-translational protein targeting and transport. Bacterial signal sequences have the same signal sequence physicochemical organization. In bacteria, the signal sequence hydrophobic core is the primary element for protein translocation through the membrane, while the N-terminal domain provides the efficiency of the process, and the C-terminal cleavage site is important for processing [38,39,40]. In mammals, the hydrophobic core is important for SRP recognition, while the N-terminus is less critical [41,42]. Signal peptidases located in the ER membrane with their active sites exposed to the lumen cleave the signal sequence at the cleavage site.

Signal sequences are distinguished by the structural elements that allow them to be efficiently recognized by their specific cytosolic targeting factors, but they are otherwise remarkably diverse, both in their amino acid composition and length. The large binding pocket of SRP is lined by methionines, which allow for a variety of sizes and shapes to fit into the pocket [41].

In bacteria, prokaryotic SRP (Ffh/4.5S RNA complex) does not target secretory proteins and targets mostly inner membrane proteins with multiple transmembrane domains (TMDs). Unlike eukaryotes, bacterial secretory proteins are targeted by SecA [43]. Bacterial SRP senses the first or second TMDs for targeting, and an average of 50–100 amino acid residues in nascent chain length are needed for recognition [44]. Ffh also targets tail-anchored membrane proteins (TAMPs) [45]. TAMPs have one transmembrane spanning domain at the carboxy-terminus that only appears after the translation has been terminated. Post-translational targeting for TAMPs is performed by Ffh if the tail-anchored portion is sufficiently hydrophobic, indicating Ffh recognizes large hydrophobic spans in proteins [46]. Ffh also requires the protein secondary structure to correctly target proteins [47].

Eukaryotic SRP requires the N-terminal signal sequence for proper targeting of secreted proteins but also identifies TMDs regardless of location [48]. SRP generally recognizes the hydrophobic stretch of amino acids in the first or second transmembrane domains of polytopic membrane proteins [49]. Similar to Ffh, eukaryotic SRP distinguishes large hydrophobic spans in proteins [47]. Unlike Ffh, however, eukaryotic SRP does not require the protein secondary structure for signal sequence recognition [47,49]. The conserved role of SRP, then, seems to be the recognition of highly hydrophobic domains, with differences arising due to the need for specificity and efficiency.

## 3. Evolution of SRP

The signal recognition particle is found ubiquitously across all three domains of life, although there is considerable variability in its structure, subunit number, and composition. The majority of SRPs consist of protein and RNA subunits. However, exceptions without RNA components are also known. The simplest version of an SRP consists of only one protein and one RNA in some bacteria. The increasing complexity of SRP characterizes the evolution of SRP from a single, evolutionarily conserved SRP54 on a short RNA to the mammalian six-subunit complex arranged on the longer and more structured RNA.

**Figure 2:**
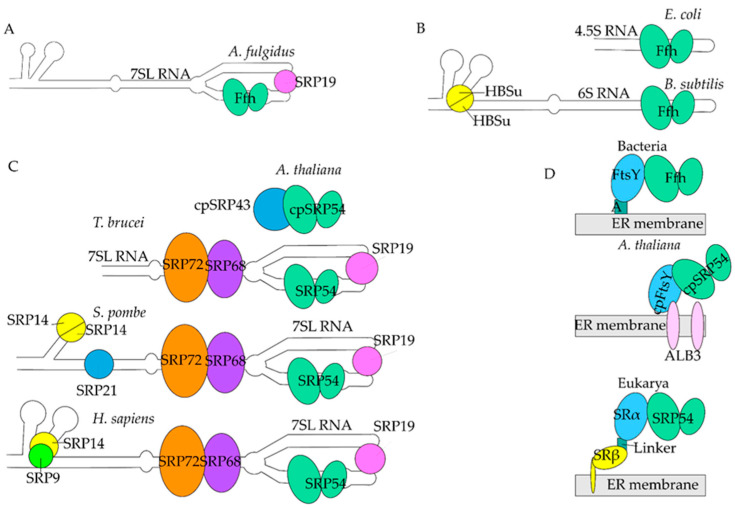
Evolution of SRP. (**A**). Archaea. Archaeal SRP has 7SL (white) with Ffh (light green ovals) and an SRP19 homolog (pink circle). (**B**). Bacteria. Bacterial SRP consists of 4.5S RNA (white) and Ffh only in *E. coli*. *B. subtilis* has HBSu subunits (yellow circle divided in half) in addition to 6S RNA and Ffh. (**C**). Eukarya. *A. thaliana* has cpSRP43 (large blue circle) and cpSRP54 (light green ovals). *T. brucei* has 7SL RNA with SRP19, SRP54 (light green ovals), SRP68 (purple oval), and SRP72 (orange oval), no sRNA76 shown for simplicity *S. pombe* has 7SL RNA, SRP19, SRP54, SRP68, SRP72, SRP21 (small blue circle), and two SRP14s (yellow circle divided in half). *H. sapiens* SRP has 7SL RNA (white), SRP19 (pink circle), SRP54 (light green ovals), SRP68 (purple oval), SRP72 (orange oval), SRP14 (yellow circle), and SRP9 (green circle). The RNA backbone and SRP54/Ffh are conserved between species. Homologs are colored similarly, excepting SRP43 and SRP21, which have no homolog in *H. sapiens*. (**D**). SRP Receptors. In eubacteria and archaebacteria, Ffh binds to FtsY (light blue oval) through a linker called A (teal square) on the ER membrane (gray rectangle). In most of the Eukarya, SRP54 binds to SRα (light blue oval), which is bound to SRβ (yellow ovals) through a linker. SRβ is embedded in the ER membrane. In *A. thaliana*, cpSRP54 binds to cpFtsY, which is bound to ALB3 (pink ovals), a chloroplast-specific receptor on the ER membrane.

The least complex signal recognition particles are among bacteria and archaea (Figure 2A,B). The SRP of gram-negative bacterium *Escherichia coli* consists of 4.5S RNA and just the single protein subunit, Ffh, a homolog of the eukaryotic SRP54 [50] (Table 1 and Figure 2). The 4.5S RNA serves as a scaffold for protein interactions, leading to Ffh rearrangement upon its binding to 4.5S RNA and FtsY (FtsY in *E. coli*, PilA in *Neisseria gonorrhoeae*) [51,52,53]. The SRP of Gram-positive bacteria such as *Bacillus subtilis* has a slightly different composition, containing longer RNA (6S RNA) and the HBSu protein, an Alu domain homolog, in addition to Ffh [54,55]. The archaebacterium SRP of *Archaeoglobus fulgidus* (Figure 2A) contains 7S RNA, an SRP19 homolog, and Ffh [55,56,57,58], and at least 10 other archaebacteria have SRP19 homologs as well (reviewed in [59]).

However, there is high variability within the Eukarya domain (Figure 2C). Plants have both cytoplasmic SRP and chloroplast-specific SRP, most likely due to the hypothesized bacterial origin of chloroplasts as stated by the endosymbiosis theory. In the endosymbiosis theory, chloroplasts originated from cyanobacteria plastids [60,61]. As a result, chloroplasts have independent DNA from the rest of the plant cell and would help explain why there are two different types of SRP in plant cells. Additionally, there are two different classifications of plants: vascular and non-vascular. Vascular plants are land plants that contain lignified tissues that conduct water and other nutrients throughout the plant, while non-vascular plants do not use this system. The vascular model plant, *Arabidopsis thaliana*, contains a chloroplast-specific SRP (cpSRP) that does not contain an RNA backbone, has chloroplast-specific SRP54 (cpSRP54), and an additional subunit cpSRP43, which may serve the same function as the noncoding RNA (Table 1, Figure 2C) [62,63]. Evolutionarily, cpSRP has more similarities in structure and amino acid composition to bacterial and archaeal SRP (Figure 2). Functionally, cpSRP co-translationally targets thylakoid membrane proteins and post-translationally targets light-harvesting chlorophyll proteins [64,65,66]. Vascular plants, such as *Arabidopsis*, seemingly have dispensed their protein-RNA binding domains in favor of protein-protein interaction between cpSRP43 and cpSRP54 in the chloroplast-specific SRP. Vascular plants also have cytoplasmic SRP. In both Arabidopsis and the tomato, *Solanum lycopersicum*, SRP54p and SRP72p have been found to be orthologs of SRP54 and SRP72, respectively [67,68]. *Solanum* also uses a modified 7SL RNA [55]. In non-vascular plants, chloroplast-specific SRP54 contains both an RNA backbone called Ffs and cpSRP43 [62]. What role this additional subunit plays in non-vascular plants remains elusive, but it may represent an evolutionary transitional step where the chloroplast-specific RNA has yet to be lost.

Other variations within Eukarya are the protists of the genera *Trypanosoma*, *Leishmania*, and *Plasmodium*. In *T. brucei* and *L. major*, the Alu domain is completely missing from the 7SL RNA and is instead replaced by a special tRNA that serves the same purpose [69,70,71] (Table 1). This tRNA, named sRNA-76 in *Trypanasoma*, is shaped like the tRNA for valine [72]. In contrast to *Trypanosoma* and *Leishmania*, *Plasmodium* does contain SRP9/14 [70,73]. All other subunits in all three protist genera are homologs to those found in *H. sapiens*. Additionally, SRP54 is essential to protists and targets polytopic membrane proteins [74]. 

One of the most studied organisms with an SRP analog to humans is yeast. The yeast 7SL RNA homologs are highly variable, from the simple fission yeast *Schizosaccharomyces pombe*, with six helices, to the more complex budding yeast *Saccharomyces cerevisiae*, with eleven helices [55]. Yeast is the only organism known to bypass the SRP targeting system by adaptation of their post-translational targeting pathways [75,76]. The yeast *S. pombe* SRP contains s1r1 RNA, an Alu domain that consists of the homodimer SRP14p, an additional subunit SRP21p, and the S domain, which contains SRP19p, SRP54p, SRP68p, and SRP72p [77] (Table 1, Figure 2).

Human SRP, one of the most complex, consists of six protein subunits arranged on a 299 nucleotide-long 7SL RNA, or SRP RNA. 7SL RNA has seven helices numbered 2–8 [78,79,80]. It is divided into two domains based on SRP function; the Alu domain, which consists of the binding region for the heterodimer SRP9 and SRP14, and the signal recognition or S domain, which consists of the binding region for SRP19, SRP54, and the heterodimer SRP68 and SRP72. All subunits are named for their molecular weights. As described in detail later, SRP9 and SRP14 function in elongation arrest, SRP19 functions to stabilize the 7SL structure, SRP54 recognizes the signal sequence, and SRP68 and SRP72 are essential for correct targeting to the endoplasmic reticulum. Additionally, 7SL RNA provides a framework for SRP68/72 to remodel 7SL, rearranging where SRP54 binds and where the GTPase domain sits [81,82,83]. Table 1 and Figure 2 summarize the different SRPs. 

SRP targets ribosome-nascent chain complexes (RNCs) to the SRP receptor. Each species has its specific SRP receptor on the cytosolic side of the ER membrane in eukaryotes or FtsY on the cytosolic side of the plasma membrane in bacteria (Table 1, Figure 2D). The eukaryotic SRP receptor (SR) consists of two subunits, SRα and SRβ, while bacterial FtsY is represented by only one protein corresponding to the eukaryotic α subunit. SRβ is anchored into the ER lumen and is tightly bound to SRα. There are three GTPases: SRP54 and SRα, which stimulate each other’s GTPase activity to mediate handover of the nascent chain to the Sec61 translocon [84,85], and SRβ, which requires a guanine nucleotide exchange factor (GEF) [86,87,88,89]. SRα predominantly stabilizes the complex, and hydrolysis of GTP is required for dissociation from SRP54 [84]. As such, the SRP54-SRα interaction is transient [90]. SRβ has six β sheets and five α-helices surrounding it, with a notable helix four that is extended [91]. The N-terminal of SRβ is buried in the ER membrane, making it an integral ER protein [92]. Mammalian SRβ is an ancient eukaryotic GTPase belonging to the Ras superfamily [89,93,94]. Purified SRβ does not show detectable GTPase activity and is purified in its GTP-bound form [86]. Although SRβ binds GTP, it has no intrinsic GTPase activity, thus requiring activation and a GEF [86]. It was shown that the β subunit of the translocon might serve as a GEF for SRβ. However, details of this process are still unknown [88]. It was also suggested that SRβ might be activated by SRP RNA [89,95]. SRα directly binds SRβ through the GTP-bound GTPase domain of SRβ [96]. The subunit α in mammals has homologs in every species discussed thus far, whereas the β subunit exists among the Eukarya domain. Since SRP exists in many different forms, it is important to consider differences in function and biogenesis.

## 4. SRP Biogenesis in Mammalian Cells

**Figure 3:**
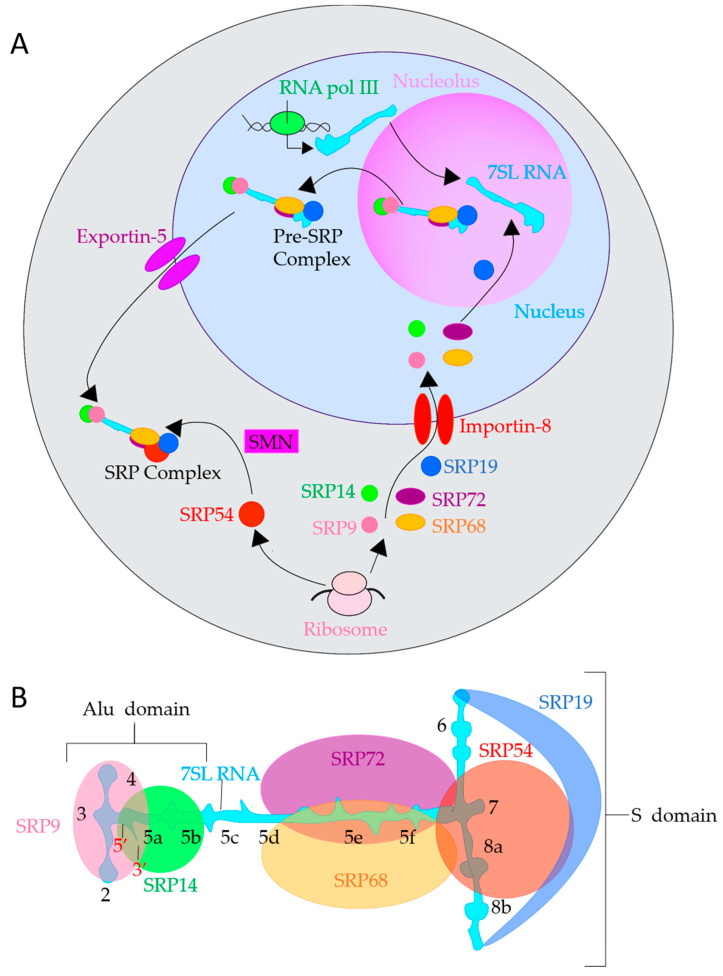
SRP Biogenesis and structure of 7SL RNA. (**A**). SRP Biogenesis in mammals. Schematic representation of SRP biogenesis: synthesis, pre-assembly, and final maturation of SRP. 7SL RNA (aqua shape) is transcribed in the nucleus (light blue region) by RNA pol III (green oval), SRP9 (salmon circle), SRP14 (green circle), SRP19 (dark blue oval), SRP54 (red circle), SRP68 (orange oval), and SRP72 (dark purple oval) are translated by ribosomes (pink ovals) in the cytoplasm (gray region). All SRP subunits except SRP54 are then imported into the nucleus by Importin-8 type transportins (red parallel ovals), with SRP19 assembling with 7SL RNA first in the nucleolus (light purple region). Then, 68/72 and 9/14 bind 7SL RNA to form a pre-SRP complex. The pre-SRP complex is exported out of the nucleus via Exportin-5 (purple parallel ovals). SMN (pink rectangle) then attaches SRP54 to the pre-SRP complex to form a complete SRP. (**B**). 7SL RNA depicted in a flat configuration to illustrate helices and binding locations of subunits. 5′ and 3′ are labeled with red numbers. Each helix is labeled with a black number.

Mammalian SRP is a multi-subunit structure with complex organization. However, there are very limited studies devoted to the assembly of the framework of SRP. A potential model for the process is shown in Figure 3A. Mammalian 7SL RNA is transcribed by RNA pol III in the nucleus (Figure 3A) [99]. Then it likely moves to the nucleolus, where it was detected by microscopy, to participate in the pre-SRP complex assembly. The majority of SRP RNA is observed in the nucleolus in the intranucleolar spaces not associated with ribosome biogenesis [100,101,102,103]. This suggests that the nucleolus has a specific function related to SRP biogenesis, which is independent of that of ribosome biogenesis. 7SL has an Alu domain (containing the AluI restriction endonuclease recognition sequence AGCT), an S domain containing an SRP54 binding site, and an SRP19 binding site (Figure 3B) [79]. Ribosomes translate mRNAs of SRP subunits in the cytoplasm, which are folded by yet unknown factors. All SRP subunits except SRP54 are then transported into the nucleus using importins. It was shown that Importin-8, transportin, and importin-β family of receptors import SRP19 in vitro [104,105]. Though other subunits have not been seen to use importin-8, it can be inferred they also use the importin-β family of receptors. SRP9/14, SRP19, and SRP68/72 colocalizes in the nucleolus [70,100]. However, SRP54 does not and only localizes in the cytoplasm as was shown for *Plasmodium falciparum* SRP54, which has approximately 48% identity with mammalian SRP [70].

After reaching the nucleus, the subunits bind to 7SL RNA in the following order: SRP19, SRP68/72, and SRP9/14, as shown in vitro [83,106,107,108]. Mammalian SRP19 binds the tetraloop between helices six and eight of 7SL RNA (Figure 3B), which collapses 7SL into a configuration that promotes further binding [102,109,110,111,112]. Helix six is positioned parallel to helix eight and rearranges 7SL to open up for SRP54 binding [113,114,115]. SRP19 maintains its structure even when not bound to 7SL RNA and rearranges a disordered loop in 7SL via reciprocal induced fit upon 7SL binding [106].

Mammalian SRP68 and 72 cohere on the opposite side of 7SL at helices five, six, and eight (Figure 3B). It appears that SRP72 binding is enhanced upon SRP68 binding [83], indicating SRP68 might bind first. Alternatively, SRP68 rearranges SRP72 into a configuration more suitable for binding. Additionally, SRP68 and SRP72 are required to export the pre-SRP complex from the nucleus in yeast [102], and a similar mechanism is assumed to exist in mammals. SRP68/72 reinforce binding between each other, but, in vitro, SRP68/72 and SRP19 bind anti-cooperatively [109].

Neither SRP9 nor SRP14 in mammals can bind 7SL RNA by itself; they heterodimerize before binding the RNA [107]. The Alu domain of 7SL RNA contains two helices and two loops that bind to SRP9/14 [108]. The complex of SRP9/14, SRP19, and SRP68/72 with 7SL RNA as a backbone creates a pre-SRP complex. Unlike budding yeast, which uses chromosome region maintenance 1 (CRM1/Exportin 1) to export 7SL RNA and its associated proteins, mammalian pre-SRP uses Exportin 5 [116]. Exportin-5 recognizes double-stranded RNA with a 3′ overhang and fits the substrate into a baseball glove-like structure with RanGTP, a small G protein that translocates RNA and proteins through the nuclear pore complex [117]. This complex of RanGTP and Exportin-5 then traffics 7SL RNA and its associated proteins out of the nucleus.

Once the pre-SRP complex is exported out of the nucleus, it then associates with SRP54, the final component of the SRP. The survival motor neuron complex (SMN) is a cell factor required for SRP54 association with 7SL RNA in mammals [118]. No other factors involving the biogenesis of SRP are known at this time, leaving several unanswered questions. Are there other factors similar to SMN involved in SRP biogenesis in the nucleolus or cytoplasm? Are there intermediate structures in the nucleolus? Which heterodimer pair is attached first? How is the quantity of SRP subunits regulated? Does SRP still form with the absence of one subunit? Further studies are required to elucidate the various aspects of the regulation of SRP.

## 5. The SRP Cycle

**Figure 4:**
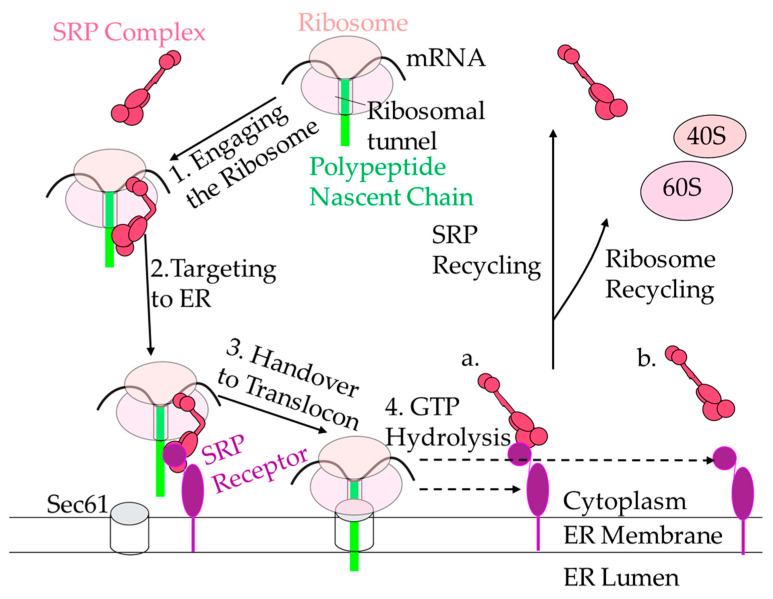
The SRP cycle. SRP recognizes the signal sequence (green rectangle) when it is exposed from the ribosomes (shown as two ovals representing small and large subunits, light pink) and binds. The SRP-ribosome-nascent-chain complex then is targeted to the ER (white) via SRα (purple circle) and SRβ (purple oval with tail-anchored portion) and handed over to the translocon. Then, either SRP and SR dissociate stepwise (**a**) or SRP and SR dissociate simultaneously (**b**). SRP then is recycled back to target other ribosomes, and the ribosomes are recycled once they reach the stop codon and dissociate.

To conduct its function, SRP is involved in a series of events called the SRP cycle. The cycle can be divided into four major steps: (1) engaging with the ribosome and recognition of the signal sequence, (2) targeting to ER, (3) engagement with the translocon, and (4) GTP hydrolysis and SRP recycling (Figure 4). There are a few hypotheses as to how SRP engages the ribosome in the first step of the SRP cycle. Walter and Blobel originally proposed that SRP scans for the hydrophobic signal sequence of the nascent polypeptide chain and only attaches to the ribosome after the nascent polypeptide chain has been exposed from the polypeptide exit tunnel at the ribosome [119,120,121,122]. However, other authors have proposed that SRP54, one of the subunits of mammalian SRP, penetrates inside the ribosomal tunnel to recognize the signal sequence and engages the ribosome before the signal sequence even leaves the tunnel exit [49,123,124,125,126]. Increasing evidence points to the possibility that SRP is already present on the ribosome when the nascent chain emerges, particularly because the binding affinity of SRP to the ribosome is in the nanomolar range and changes as the signal sequence emerges [124,126,127]. However, it is not completely understood how SRP distinguishes between ribosomes translating secretory and cytosolic proteins in this case. Are there specific ribosomes specialized to synthesize proteins for transport? Does SRP bind only specialized ribosomes that are translating secretory and membrane proteins? These are very important fundamental questions to be answered in the future.

After recognition of the signal sequence by SRP54, SRP engages with the ribosome and temporally stalls elongation of the nascent polypeptide chain (Step 1, Figure 4). During this event, SRP changes its conformation, positioning SRP9/14 proteins forming the Alu domain near the elongation factor binding site of the ribosome, physically preventing synthesis of polypeptides [128,129]. SRP9/14 also appears to start positioning the ribosome towards the translocon during elongation arrest [130]. Truncating SRP14 effects elongation delay activity and restructures the 7SL RNA, indicating the Alu domain and the conformation of 7SL RNA are crucial in maintaining elongation arrest [131]. Evolutionarily, it appears that the Alu domain is unnecessary for SRP function, as seen by the lack of an Alu domain in the chloroplast-specific SRP in non-vascular plants, eubacteria, and archaebacteria. The Alu domain of eukaryotes does not influence translocation capabilities [132]. However, despite the Alu domain not being considered essential for SRP function, SRP9/14 and its homologs aid in targeting efficiency [133]. The Alu domain functions by causing elongation delay, which causes ribosome pausing, reducing the need for multiple SRP per mRNA transcript [134]. This helps explain why the concentration of SRP is approximately 50 times less than that of ribosomes in eukaryotic cells [126]. Additionally, elongation delay allows the SRP receptor time to transfer its ribosomal cargo to the translocon. There are about two-fold fewer receptors than co-translational translocons on the ER surface [115], so without elongation delay, the receptor would quickly reach saturation and stall the cycle. The temporal elongation arrest also helps to prevent synthesis and accumulation of potentially hazardous proteins in the cytosol, providing time for ribosome nascent-chain targeting to the ER. 

Targeting the SRP receptor on the ER membrane is the next step in the SRP cycle (Step 2, Figure 4). SRP54 associates with the signal recognition particle receptor (SR) using its G domain, which has intrinsic GTPase activity. SR, in general, has a higher affinity for the ribosome than SRP; the SRP-SR interaction is predicted to improve the accuracy of targeting to the ER, whereas SR-ribosome interactions aids in the speed of targeting to the translocon [135]. SRP then hands over the ribosome and nascent chain to the translocon (Step 3, Figure 4) [136,137].

Once SRP has delivered its ribosomal cargo to the translocon, it enters a post-handover state (Step 4, Figure 4). There are two mechanisms of action through which the post-handover state may occur: simultaneously or stepwise. GTP hydrolysis occurs simultaneously between SRβ and the ribosome, and SRα and SRP54, hydrolyzing GTPs to dissociate from the ribosome and nascent chain. In stepwise hydrolysis, the dissociation of SRβ and the ribosome, and SRP54 and the SRα, happen in two steps. SRα and SRP54 hydrolyze GTP to be released from each other and the signal sequence once the translocon is present; then SRβ hydrolyzes GTP, but it is unclear how GTP hydrolysis happens or whether SRβ is dissociating from the ribosome (Step 4, Figure 4) [137,138,139,140]. In both scenarios, the translocon promotes and regulates the GTPase activity of SRP-SR [89,95,98,137], and SRβ is required for the signal sequence release from the ribosome to the translocon [141]. Recent publications have not been able to elucidate which mechanism is more accurate as the structure of the post-handover state is still unknown. SRP is then recycled to start the cycle again.

## 6. Structure and Function

**Figure 5:**
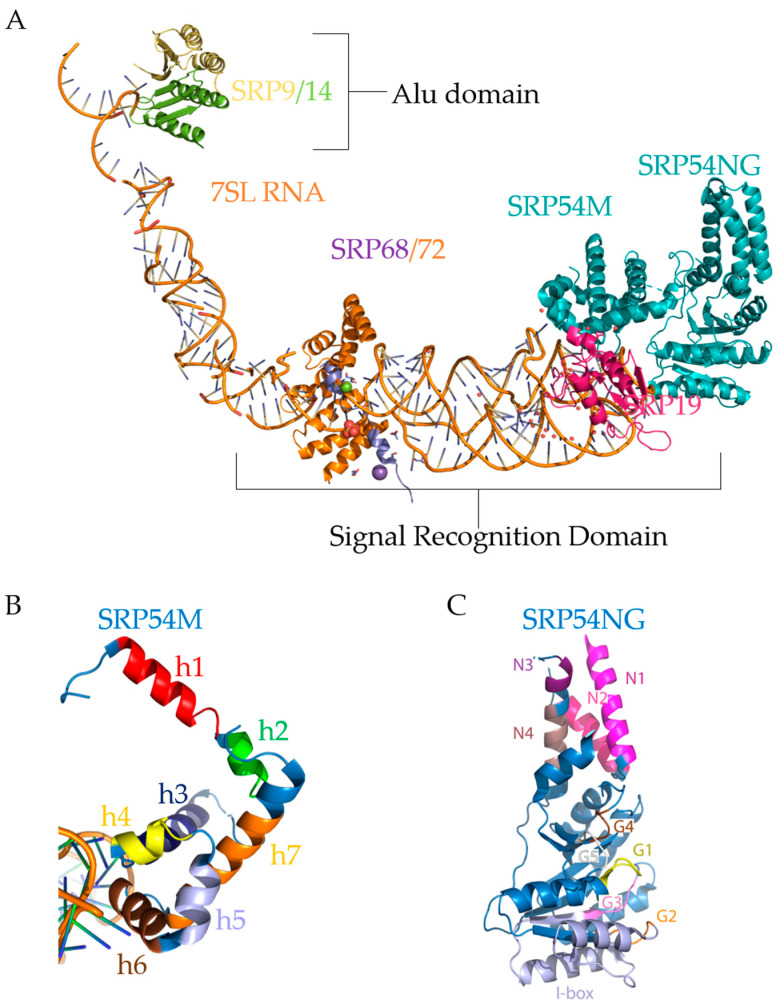
Mammalian SRP. (**A**). Illustration (not an actual structure) of a composite SRP complex constructed using PyMol software. The image was created in PyMol [145] by the using of coordinates for SRP subunit structures from the Protein Data Bank (PDB) [146] with the following PDB IDs and references to corresponding publications: 5WRV and 5WRW [144], 1RY1 [129], and 1MFQ [113]. SRP Subunits and RNA were aligned using PyMol, which aligns based on superposition then refines the fit. 1RY1 was used to align all subunits on 7SL RNA (orange double helix). The Alu domain and the S domain are labeled. SRP9 (yellow), SRP14 (green), 7SL RNA (orange helix), SRP68 (purple), SRP72 (orange), SRP19 (pink), and SRP54 (teal) are marked. (**B**). The SRP54M Domain. Pymol representation of the SRP54M domain on 7SL RNA (orange double helix) using the PDB coordinates 1MFQ [113]. The M domain consists of the alpha-helices: h1 (red), h2 (green), h3 (dark blue), h4 (yellow), h5 (periwinkle), h6 (brown), and h7 (orange). Helices 1–3 bind the signal sequence. Helices 4–7 create a tunnel through which a signal sequence can be recognized. (**C**). PyMol representation of the SRP54 NG domain using the PDB coordinates 5L3Q [143]. The N domain consists of αN1 (pink), αN2 (hot pink), αN3 (purple), and αN4 (gray). The G domain consists of G1 (yellow), G2 (orange), G3 (purple), G4 (brown), and G5 (white). The SRP54NG domain mediates SRα binding and GTP hydrolysis.

The structure of the whole mammalian SRP complex associated with the ribosome was solved by cryo-EM [123,129]. Several structures of the separate SRP subunits are also currently available. Cryo-EM and X-ray structures for SRP9/14 and SRP19 have been solved in *H. sapiens* by [106,108,128]. SRP54 from archaebacteria, eubacteria, dogs, mice, humans, and plants have all been solved by various authors [79,142,143]. Human SRP68 and SRP72 have only been partially solved [83,144]. Using PyMol software [145], we constructed an illustration of the composite SRP by taking the PDB coordinates of Cryo-EM and X-ray structures of SRP54NG domain and 7SL RNA from Protein Data Bank entry [146], 1RY1 [129], SRP19 and SRP54M domain from 1MFQ [113], and the protein-binding and RNA-binding domains of SRP68 and SRP72 from 5WRV and 5WRW [144], respectively, shown in Figure 5A.

Eukaryotic SRP is 230–240 Å in length as measured by scanning transmission electron microscopy and is not much smaller than the eukaryotic ribosome at 250–300 Å [147,148]. Since its discovery in the 1980s [149], SRP research has focused on the functional characterization of the different subunits of SRP and the SRP cycle. When the core of SRP (SRP54) has recognized a signal sequence and attached to a ribosome, then it is in an L-shaped conformation, as shown in Figure 5A. Notably, the figure shows three different regions of SRP: the Alu domain, located towards the top left, which consists of the heterodimer SRP9/14; the linker region, consisting of mostly 7SL RNA and parts of SRP68/72; and the S domain, consisting of the heterodimer SRP68/72, SRP19, and SRP54.

Structurally, SRP9 acts as a clamp on the 5′ and 3′ ends of 7SL RNA [150], shown in Figure 3B and Figure 5A in the Alu domain. Mammalian SRP9/14 has an α-β-β-β-α tertiary motif that defines the Alu binding motif [78]. The heterodimer SRP9/14 forms a saddle, with four α-helices in the middle and a concave β-sheet surface lined with positive residues [78]. This saddle physically occludes the inter-ribosomal-subunit space at the A-site [128,129]. Interestingly, 7SL RNA and 5S ribosomal RNA, which mediates between the peptidyl-transferase site and the GTPase center, may interact with the same targets due to sequence homology [151]. Since 7SL shares homology, it is likely some part of 7SL may sit near the P-site, and indeed the Alu domain sits on the A-site near the P-site as shown by its X-ray structure [129].

A central region of SRP9 and three regions of SRP14 (C-terminus, and the loop regions between β-sheets containing amino acids 33–43 and 44–55) are required for heterodimerization [152]. SRP14, therefore, appears to be more sensitive to structural mutations that affect dimerization than SRP9; however, the RNA can still interact with mutated SRP14 to stabilize it [152]. The N-terminus of SRP9 is required for RNA-binding, whereas the first loop region containing amino acids 33–43 in SRP14 is required for RNA-binding. It is unknown whether eliminating the Alu domain or rearranging the 7SL RNA affects the S domain subunits of SRP, though it is unlikely due to the physical separation of the Alu and S domains.

Less is known about the second heterodimer pair SRP68/SRP72, located in the linker/S domain region of SRP, as shown in Figure 5A. SRP72 has a protein-binding domain (PBD) and a ribosome-binding domain (RBD) [83]. The SRP72 protein-binding domain contains a tetratricopeptide repeat, a motif that consists of 34 degenerate amino acid repeats arranged into 3–16 tandem helices assembled into a superhelical structure [153], and facilitates binding of SRP68 into the multiprotein complex SRP. The SRP72 ribosome-binding domain binds the 5e/f loop of 7SL (Figure 3B) [83]. SRP68 has a tetratricopeptide repeat that binds the SRP RNA and bends it, allowing the 5f loop to contact the ribosome [82] to coordinate it. Interestingly, SRP72 has a C-terminal contact between SRP72-RBD and the ribosome that is cleaved during apoptosis [154], which may indicate SRP72 functions more as a structural/coordinating protein. Little is currently known about the SRP68/72 role in SRβ binding and transfer to the translocon. Additionally, SRP68 mediates SRP72 binding as a heterodimer, and SRP68 remodels 7SL RNA [82]. Physiologically relevant mutations are seen in the non-crystallized regions of SRP68/72, but it is not yet determined whether the heterodimer aids in targeting or functions more as a coordinating factor for the ribosome.

SRP54 binds 7SL at the end of the S domain, shown in Figure 5A, and part of the structure of SRP54 was crystallized in 1999 by Clemons et al. [155]. They demonstrated that SRP54 consists of two domains: the methionine-rich (M) domain (Figure 5B), which recognizes the signal sequence of SRP-dependent secretory and transmembrane proteins; and the N-terminal GTPase (NG) domain (Figure 5C), which docks with the signal recognition particle receptor (SR) subunit α. The methionine-rich (M) domain (Figure 5B) consists of seven alpha-helices; helices 4–6 bind 7SL RNA [155]. Helices 1–3 (red, green, and dark blue helices, respectively, in Figure 5B) are involved in signal sequence binding, with a finger loop between helices 2 and 3 [155]. This finger loop, depicted under helix 7 (orange) in Figure 5B, may be used to penetrate the ribosomal tunnel to scan for the signal sequence. The rest of the helices (4–7 corresponding with yellow, periwinkle, brown, and orange helices, respectively, in Figure 5B) of the M domain forms a hydrophobic pocket for the signal sequence to translate through, which is sufficient and necessary to recognize signal sequences and TMDs [156,157]. The hydrophobic pocket, or tunnel, can clearly be seen in Figure 5B. However, eliminating M domain binding to the RNA also abolishes the recognition of signal sequences [158], indicating that, while helices 4–6 (yellow, periwinkle, brown) are mainly for anchoring, without them, the tunnel cannot form, leading to signal sequences not being recognized.

The G domain of SRP54 has five GTPase conserved elements—G1 through G5, as shown in Figure 5C. G1 (yellow helix in Figure 5C) consists of the Walker A/P-loop motif, which creates an anion hole for β-phosphate binding and is the active site for GTPase activity [159]. G2 (orange loop) is the start of the insertion box (periwinkle helices), or “I box”, the SRP-specific insertion box protein motif that mediates GTP hydrolysis [143] that is absent in other GTPases. G3 (purple loop) is also involved with magnesium coordination with water, and the arginine at the end coordinates the third (γ-) phosphate of GTP by stabilizing the transition state of the GTPase reaction [143,159,160]. G4 (brown loop) and G5 (white loop) help coordinate and bind the guanine nucleotide of GTP. G5 is at the end of the G domain and forms a pocket for the guanine to sit [143]. The N-terminal GTPase domain also interacts with 7SL RNA and SR [161]. 

The subunit SRP19, and the long, noncoding RNA backbone, 7SL RNA, indirectly assist the SRP cycle. Their function is related to the biogenesis of SRP, as described previously and as illustrated in Figure 3A. 7SL RNA also has a function outside of SRP. The Alu domain contained in 7SL is the most abundant retrotransposon in the human genome [162], and 7SL RNA is associated with cytoskeletal proteins in blood [163].

## 7. RAPP and SRP

**Figure 6:**
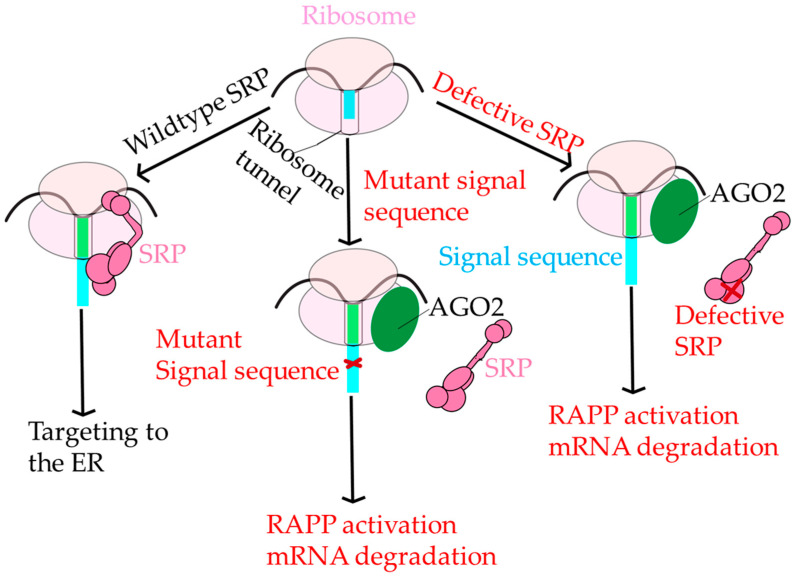
Regulation of Aberrant Protein Production. Under normal conditions, SRP (dark pink) engages the ribosome (pink ovals) and correctly targets polypeptide nascent chains with signal sequences (light blue rectangle) to ER for further protein transport. However, if SRP cannot recognize the signal sequence due to a critical mutation in the signal sequence (red x), then AGO2 (green circle) binds to the RNC complex instead of SRP initiating the RAPP pathway and leading to mRNA degradation. There is a third possibility; if SRP is defective and unable to recognize the signal sequence, then the RAPP pathway is activated as well.

Besides SRP’s role in co-translational targeting, there is accumulating evidence that SRP protects the mRNA transcripts of SRP-dependent proteins from degradation [164,165,166,167]. If SRP cannot recognize the nascent chain of SRP-dependent proteins, then a quality control mechanism called the Regulation of Aberrant Protein Production (RAPP) is activated, and the mRNA of the protein is degraded [164]. Figure 6 illustrates RAPP. However, details of the RAPP mechanism are not known, and the enzyme(s) degrading the mRNA has not been identified. Although the RAPP mechanism is not understood, there are many RAPP substrates identified, including proteins with disease-causing mutations in humans [165,167]. Many human diseases, including some forms of frontotemporal lobular degeneration, were associated with pathological RAPP activation [165,166,167]. This suggests that the molecular mechanisms of these disorders are through RAPP pathway activation. 

RAPP is functionally distinct from other protein quality control pathways, including endoplasmic reticulum-associated protein degradation (ERAD) and the unfolded protein response (UPR), reviewed in [168,169]. In RAPP, signal sequences with mutations that adversely affect the hydrophobicity of the H domain do not associate with SRP54 and instead become close in proximity to Argonaute2 (AGO2) [164]. RAPP is also distinct from other mRNA quality control mechanisms in the cell. For a review of mRNA quality control mechanisms, see [168]. It is unknown whether there is any overlap between proteins that sense and trigger RAPP and other mRNA degradation pathways. The role AGO2 plays in RAPP is ambiguous, and, in general, the RAPP pathway is poorly understood. The major role of AGO2 is as a part of the RNA-induced silencing complex, or RISC, which uses micro RNAs (miRNAs). This may indicate an alternative suppressive role of AGO2 in RAPP since RAPP does not require miRNAs or AGO2 ribonuclease H (slicer) activity. AGO2 has also been identified as a translational repressor working independently of TRIM71, a ubiquitin ligase that represses mRNA function [170]. Ultimately, AGO2 appears as a sensor of RAPP, suggesting the independence of RAPP from other major mRNA degradation pathways and the establishment of SRP as a complex involved in the protection of mRNA.

## 8. SRPassing Co-Translational Targeting

SRP has evolved from a single protein subunit complex mediating the co-translational pathway in early forms of archaebacteria and eubacteria to a complex, six-subunit protein with a long, noncoding RNA backbone. SRP has multiple functions in prokaryotes and eukaryotes: from targeting polytopic membrane proteins only in prokaryotes to the targeting of membrane and secretory proteins in eukaryotes, and the recently discovered function of mRNA protection in eukaryotic SRP.

mRNA protection is still relatively undefined in SRP; RAPP involves the lack of recognition by SRP54 and degradation of mRNA and the involvement of AGO2. However, each protein’s complete role in RAPP is still elusive. Structures of most of the SRP subunits have been resolved; however, SRP68 and SRP72 still have un-crystallized regions and have a poorly defined role in the SRP cycle. Overall, despite being discovered in 1980, SRP still has quite a few mysteries left to be resolved and has surpassed its originally defined function in co-translational targeting.

## Figures and Tables

**Table 1:** Evolutionary comparison of different SRPs to *H. sapiens* SRP.

Species	SRP RNA	Alu Domain		S Domain				Additional Subunits	Receptor	Source
*Homo sapiens*	7SL RNA	SRP9	SRP14	SRP19	SRP54	SRP68	SRP72	-	SR	[97]
*Trypanosoma brucei*	7SL RNA	sRNA76		SRP19	SRP54	SRP68	SRP72	-	SR	[69,74]
*S. pombe*	s1r1 RNA	SRP14p	SRP14p	SRP19p	SRP54p	SRP68p	SRP72p	SRP21p	SR	[77,98]
*Archaeoglobus fulgidus*	7S RNA	-	-	SRP19	Ffh	-	-	-	FtsY	[55,56,57,58]
*Escherichia coli*	4.5S RNA	-	-	-	Ffh	-	-	-	FtsY	[50]
*Bacillus subtilis*	6S RNA	HBSu	HBSu	-	Ffh	-	-	-	FtsY	[54,55]
*Arabidopsis thaliana*	-	-	-	-	cpSRP54	-	-	cpSRP43	cpFtsY	[57,58,62,63]

## Data Availability

Not applicable because it is a review.
